# Retrospective Case-Control Study of Extended Birth Perineal Tears and Risk Factors

**DOI:** 10.7759/cureus.57132

**Published:** 2024-03-28

**Authors:** Mohammad Dendini, Sara K Aldossari, Hydar A AlQassab, Othman O Aldraihem, Amwaj Almalki

**Affiliations:** 1 Urogynecology and Reconstructive Pelvic Surgery, King Abdulaziz Medical City, Ministry of National Guard Health Affairs, Riyadh, SAU; 2 Medicine, College of Dentistry, King Saud Bin Abdulaziz University for Health Sciences, Riyadh, SAU; 3 Medicine, King Saud University, Riyadh, SAU; 4 Biostatistics and Epidemiology, King Abdullah International Medical Research Center, Riyadh, SAU

**Keywords:** risk factors, epidural anesthesia, perineal tear risk factors, instrumental delivery, extended perineal tear, perineal injury

## Abstract

Background: A perineal tear is a rupture of the skin or muscle between the vagina and anus (perineum). A third-degree tear is one type of extended perineal tear (EPT), and it involves the penetration of the anal sphincter muscle. Another type of EPT is a fourth-degree laceration, which penetrates deeper into the lining of the anus or rectum. The stretching of the perineum during childbirth may result in perineal trauma. Invasive surgical interventions are required for the treatment of EPTs. For this reason, the reduction of their incidence can be achieved by fully comprehending the risk aspects associated with them.

Objective: The aim of this study is to contribute to the body of knowledge by providing insight into the various risk factors that are associated with extended perineal trauma. By following this path, this study aims to contribute to and advance Saudi Arabia’s development of evidence-oriented obstetric care recommendations.

Study design: The current study is a case-control study where a review of 5000 vaginal delivery records was carried out between March 1, 2018, and March 19, 2023. For the study, cases (n = 71) were female patients who had documented greater than second-degree perineal injury. The control group (n = 238) was randomly chosen from women who had vaginal delivery but with less or equal to a second-degree perineal laceration. For each patient, we reviewed medical and obstetrics records for the following characteristics: age of diagnosis, gestational age, parity, labor induction, second-stage labor duration, mode of delivery, infant birth weight, epidural use and episiotomy indication, healthcare worker (HCW) experience, and APGAR (appearance, pulse, grimace, activity, and respiration) score.

Results: From the 5000 births analyzed, the cases were 71 patients (1.42% of 5000 births). The mean age at diagnosis in our sample was (28.05 ± 4.66 years). The study's results showed that the following variables significantly affected the occurrence of EPTs: birth weight, labor durations, parity, HCW experience, and mode of delivery. The odds for tears were 3.69 (95% CI: 0.156-0.469) higher in nulliparous patients relative to multiparous patients. Instrumental deliveries resulted in more tears than non-instrumental deliveries with an odds ratio (OR) of 5.901 (95% CI: 2.443-14.525). This study also found that prolonged second-stage labor seems to be associated with an increased risk of perineal damage. HCW experience was looked at in relation to the increased incidence of EPTs, which showed that midwives had a lower incidence rate than physicians with an OR of 2.25 (95% CI: 1.169-4.366). Epidural usage has also been significantly associated with a lesser incidence of perineal tears, which indicates that using epidural could protect against the occurrence of EPTs.

Conclusion: In conclusion, the occurrence of perineal lacerations could be prevented during childbirth by taking preventative measures and having more precise judgments. Epidural was a protective factor in our study against the incidence of extensive perineal tearing. Furthermore, as compared to midwives, our study showed that the majority of EPTS occurred in cases of physicians (residents/consultants). Further research, proper documentation, and the development of evidence-based guidelines are needed to improve perineal care and reduce EPT incidence.

## Introduction

The perineum is the inferior outlet of the pelvis; it is diamond-shaped and at risk of damage during vaginal delivery. A perineal laceration is an injury to the skin or muscle between the vagina and anus (perineum). The severity of perineal lacerations varies, and they are often classified from first to fourth degree. Extended perineal tears (EPTs) are also known as third- and fourth-degree perineal tears. Third-degree tears extend into the muscle that controls the anus (the anal sphincter). Fourth-degree tears extend further into the lining of the anus or rectum. During childbirth, the perineum is stretched as the baby's head passes through the birth canal. Perineal trauma can occasionally arise from this stretching, causing tears or abrasions in the perineal tissue [1]. Approximately 80% of women experience perineal tears during childbirth, with a higher prevalence among primiparous women compared to multiparous women [2,3]. Among primiparous women, the reported rate of second-degree perineal tears ranges from 35.1% to 78.3%, while among multiparous women, it ranges from 34.8% to 39.6% [2-4]. Third- and fourth-degree tears occur in approximately 5.1% to 8.3% of primiparous women and 1.8% to 2.8% of multiparous women [2-6].

It is important to note that obstetric anal sphincter injuries (OASIS) are the leading obstetric risk factor for the development of anal incontinence in women [7]. Therefore, these tears require special attention. In Saudi Arabia, there is a lack of studies regarding the risk factors of EPTs. This study offers further evidence regarding previously studied perineal tear risk factors, Furthermore, it also emphasizes the role of some other neglected factors such as the role of health care worker (HCW) experience on the occurrence of EPTs. This study also aims to identify some protective factors such as epidural injections. Other risk factors such as episiotomy, the mode of delivery, and parity have been associated with the incidence of extended perineal tears; however, after the adjustment of the statistical models, only the mode of delivery, parity, HCW experience, and epidural usage have had relatively significant results. Understanding the role of the risk factors studied in this research can improve current guidelines of obstetric care regarding the protection and prevention of extended perineal tears. This study aims to contribute significantly to the existing body of knowledge by providing new insights into the risk factors associated with perineal injuries. By doing so, the goal is to inform and enhance the development of evidence-based guidelines for obstetric care in Saudi Arabia. Through this research, the overall quality of care provided to pregnant women is sought to be improved, ultimately promoting better maternal health outcomes.

## Materials and methods

Study area

This research is a case-control study with a 1:3 case-to-control ratio. Samples including both the EPT group and the unaffected group were selected from all vaginal childbirths at King Abdulaziz Medical City (KAMC), Saudi Arabia, Riyadh. The sample was collected from previous patients' medical records registered between March 2018 and March 19, 2023.

KAMC Is one of Saudi Arabia's widely recognized and comprehensive medical cities. The medical facility offers a variety of services ranging from basic primary care and public health to advanced tertiary care. KAMC also provides an extensive selection of therapeutic options such as mental health services, acute care, rehabilitation, and research publication services.

In this research, obstetric patients who are females, aged between 18 and 55, were included. The Department of Obstetrics and Gynecology supported this research by providing the authors with sufficient data to carry out the study. This study was approved by the Institutional Review Board for Human Research at King Abdullah International Research Medical Center (KAMARC), Riyadh, Saudi Arabia.

Study subjects

During the research period about 13000 vaginal births at more or equal to 26 weeks of gestation were reported. The patients with data that were insufficient or involving breach/twin deliveries and deliveries that happened outside of the hospital grounds were all excluded from the study. Thus, only 5000 charts qualified for the examination.

Chart audits were examined for each case (n=116) of perineal lacerations that extended beyond a second-degree tear. A total of 71 patients diagnosed with EPT were kept for analysis after the exclusion of files with absent values. For unaffected subjects (n=507), they were selected randomly out of obstetric patients who gave birth vaginally and did not sustain more than a second-degree perineal injury. After the removal of samples with missing values, there were 238 control subjects available for analysis. The selection of the EPT group and the unaffected group was finalized within the same time frame.

Data collection

Information containing the date and time of delivery, the mother’s age, the gestational age, the parity, the method of delivery (instrumented or non-instrumented), the degree of laceration (none to fourth), episiotomy indication (yes/no), the length of the second stage of labor (minutes), the weight of the fetus (grams), the experience of the healthcare worker (midwife/physician), the APGAR (appearance, pulse, grimace, activity, and respiration) score (from 1 to 9), and the induction of labor (yes/no), all were collected from medical records through direct data entry into a computerized system. The final complete data set included 238 control individuals and 71 cases. However, records of labor duration were found in only 49 out of the 71 patients diagnosed with EPTs.

Data analysis 

Data analysis was done using SAS Software Version 9.4 (SAS Institute Inc., Cary, North Carolina, United States). Categorical variables including labor induction, indication for episiotomy, delivery mode, epidural usage, healthcare provider experience, and APGAR score were calculated using frequencies and percentages. Continuous variables including gestational age, labor duration, mother’s age, and baby birth weight were represented using mean and standard deviation (SD). 

The association between continuous and two-level categorical variables was determined using the Mann-Whitney test. Furthermore, Fisher’s exact test or Chi-square test was used to compare categorical variables, as appropriate. 

The p-values of all variables were two-tailed. Static significance is defined when the p-value is less than 0.05. Unconditional logistic regression was used to evaluate the relative chances of perineal injuries during childbirth in association with instrumental and non-instrumental deliveries. Other additional factors that were predictive are parity, mother’s age, epidural use, episiotomy performance, and infant birth weight. An adjusted logistic regression was used for a multivariate analysis adjusted for independent covariates such as the mode of delivery, parity, epidural injection, and infant birth weight.

## Results

During the study period, a total of 5,000 vaginal deliveries were reviewed. The total incidence of lacerations greater than second-degree (EPT group) was found to be 1.42% (n = 71 lacerations). In our study, we tested the association between the mode of delivery (instrumental/spontaneous vaginal delivery), parity (nulliparous/multiparous), maternal age, birth weight, episiotomy indication, HCW experience (physician/midwife), APGAR score, gestational age, delivery duration at the second stage of labor, and epidural use. Instrumental deliveries were employed in 5.83% (18 out of 238 control subjects). However, Instrumental deliveries were used in 8.74% of the extended perineal tears (27 out of 71 patients). A total of 27.18% of patients were indicated for episiotomy and were not diagnosed with EPTs; however, 13.92% of the total sample were indicated for episiotomy and acquired EPTs. In contrast, the largest percentage of patients who were not indicated for episiotomy was 49.84% and did not acquire third- or fourth-degree lacerations. The patients who had been administered epidural injections had the lowest incidence of EPTs, which was 8.41%, whereas, among the undiagnosed group, epidural usage frequency was quite similar. Multiparity was frequently associated with normal EPT-free delivery with a percentage of 55.34%. A total of 9.39% of women who were multiparous had the diagnosis of EPTs; however, 13.59% of women were nulliparous. Labors that were not induced physically or medically had the highest incidence of EPTs, which was 16.50%, whereas, 6.47% of patients who had induced labors had the outcome of severe perineal tears. Furthermore, the APGAR score for infants of 67 patients out of the 71 diagnosed with EPTs was within normal limits. The APGAR score was found to be lower than 6 in 1.29% of the EPT group (Table 1).

**Table 1:** Baseline characteristics n (%) for categorical variables; mean ± (SD) for continuous variables SD: standard deviation; APGAR: appearance, pulse, grimace, activity, and respiration

Characteristics	Extended perineal tear: N = 71 (22.98%)	No tear: N = 238 (77.02%)
n (%)		
Mode of delivery: instrumented	27 (8.74%)	18 (5.83%)
Mode of delivery: non-instrumented	44 (14.24%)	220 (71.20%)
Episiotomy: yes	43 (13.92%)	84 (27.18%)
Episiotomy: no	28 (9.06%)	154 (49.84%)
Epidural use: yes	26 (8.41%)	117 (37.86%)
Epidural use: no	45 (14.56%)	121 (39.16%)
Parity: nulliparous	42 (13.59%)	67 (21.68%)
Parity: multiparous	29 (9.39%)	171 (55.34%)
Induced labor: yes	20 (6.47%)	92 (29.77%)
Induced labor: no	51 (16.50%)	146 (47.25%)
Healthcare provider experience: midwife	13 (4.21%)	80 (25.89%)
Healthcare provider experience: physician	58 (18.77%)	158 (51.13%)
APGAR score: 1-6	4 (1.29%)	10 (3.24%)
APGAR score: 7-9	67 (21.68%)	228 (73.79%)
mean ± (SD)		
Infant birth-weight (g)	3169.04 ± 425.68	3042.04 ± 476.35
Maternal age (years)	28.06 ± 4.67	30.07 ± 5.82
Gestational age (weeks)	39.27 ± 1.29	38.32 ± 1.71
Labor duration (minutes)	52.55 ± 56.22	46.07 ± 80.37

The mean infant birth weight for infants born to patients diagnosed with EPTs was 3169.04 grams, with an SD of 425.68. Furthermore, the control group had a mean infant birth weight of 3042.04 grams, with an SD of 476.35. The mean labor duration for patients diagnosed with EPTs was 52.55 minutes, with an SD of 56.22. Furthermore, the control group had a mean labor duration of 46.07 minutes, with an SD of 80.37. The mean gestational age of the control sample was 38.32 with an SD of 1.71. Moreover, the mean gestational age of patients diagnosed with EPTs was 39.27 weeks, with a standard deviation of 1.29, and the mean maternal age at the time of diagnosis in our sample was 28.06 years with a 4.67 SD. For details about the characteristics of the study population, please refer to Table 1.

A group of various obstetric parameters was associated with third- and fourth-degree perineal tearing. Only four parameters had statistically significant results after adjustment with the other variables, such as nulliparous mothers, instrumental mode of delivery, and epidural usage. The first predictor that yielded significant results after adjustment was the mode of delivery. The mode of delivery had an OR prior to adjustment of 7.499 (95% CI: 3.804-14.780) and after adjustment of 5.901 (95% CI: 2.443-14.525) and a p-value of less than 0.0001. The second one is parity where nulliparity was more prevalent in the EPT group versus the control group. Parity had a significant p-value (<0.0001) and an odds ratio of 3.696 (95% CI: 2.1302-6.4138) prior to adjustment. Other variables such as Infant birth weight and epidural usage were also associated with the outcome of EPTs. Furthermore, the p-value of epidural anesthesia was insignificant prior to model adjustment; however, after adjustment, it showed a highly significant p-value (0.0145) and OR 0.404 (95% CI: 0.190-0821) (Table 2).

**Table 2:** p-value and odds ratio of the predictors The table shows the p-values and odds ratio of variables pre and post model adjustment. A p-value lower than 0.05 is significant; odds ratios equal to 1 are insignificant, significantly lower than 1 is an indicator of a protective variable, and significantly higher than 1 is a causative variable; 95% CI: 95% confidence interval; APGAR: appearance, pulse, grimace, activity, and respiration

	Unadjusted model			Adjusted model
Predictor	p-value	Odds ratio (95% CI)	p-value	Odds ratio (95% CI)
Parity (nulliparity vs. multiparity)	<0.0001	3.696 (2.1302-6.4138)	0.0060	2.919 (1.357 - 6.321)
Delivery mode (instrumented vs. non-instrumented)	<0.0001	7.499 (3.804-14.780)	< .0001>	5.901 (2.443 - 14.525)
Epidural used (yes vs. no)	0.0644	0.598 (0.346-1.031)	0.0145	0.404(0.190-0.821)
Health worker experience (physician vs. midwife)	0.0153	2.259 (1.169-4.366)	0.0442	0.390 (0.156-0.976)
Birth weight (g)	0.0456	1.001 (1-1.001)	0.0045	1.001 (1.000- 1.002)
Episiotomy (yes vs. no)	0.0002	2.815 (1.632-4.856)	0.3960	0.677 (0.275- 1.665)
Gestational age (weeks)	0.0403	1.223 (1.009-1.483)	0.1294	1.256 (0.936- 1.686)
Induction of labor (yes vs. no)	0.1087	0.622 (0.349-1.111)	0.0584	0.447 (0.194- 1.029)
APGAR score (1-6 vs. 7-9)	0.6117	1.361 (0.414-4.480)	0.3668	2.007 (0.442- 9.114)

Unadjusted gestational age and episiotomy, both had significant p-values. However, after model adjustment, the p-values of these variables showed insignificant results (Table 2).

In the unadjusted model, delivery by physicians was related to a substantially increased risk of perineal lacerations; it had a p-value of 0.0153 and an OR of 2.259 (95% CI: 1.169-4.366). This showed that the HCW experience results were significant prior to model adjustment. After adjustment of the statistical model, HCW experience was also significant with a p-value (0.0442) and an OR of 0.390 (95% CI: 0.156-0.976) (Table 2).

Labor duration prior to model adjustment had an insignificant p-value of (0.5904) and an OR of 1.001 (95% CI: 0.997-1.005). All in all, prior and post model adjustment these findings suggest that longer birth duration had no significant effects on the occurrence of extended perineal tear. As for episiotomy indication (yes/no), a chi-square test was done and the p-value obtained was 0.0002. In the unadjusted model, the OR was 2.815 (95% CI: 1.632-4.856). Post model adjustment, both the p-value and OR yielded insignificant results. Moreover, the gestational age had a slightly significant p-value (0.0403) and an OR of 1.223 (95% CI: 1.009-1.483) before model adjustment; however, after model adjustment, both values were insignificant. Variables such as low APGAR score, second stage of labor duration, and maternal age had no significance in relation to EPT occurrence post or prior model adjustment. Birth weight p-value was significant prior to and post adjustment; however, the odds showed that it does not affect positively or negatively the incidence of EPTs (Table 2).

## Discussion

Previous research identified several risk factors that were found to significantly increase the chances of having extensive perineal lacerations, such as nulliparity, higher infant birth weight, induction of labor, maternal age over 25 years, instrument-assisted deliveries, and prolonged second stage of labor [8-10]. Operative delivery and episiotomy together have been associated with a higher risk of EPTs [11,12]. Christianson et al. reported that among the different methods of operative deliveries, forceps were associated with a greater risk of EPTs [13]. However, there is conflicting data regarding the role of episiotomy in relation to EPTs. Eskandar et al. reported that 65% of perineal tear risks were decreased by the performance of mediolateral episiotomy, which showed an absolute risk reduction of 1.1% [14]. Furthermore, past studies demonstrated that episiotomy was linked to lower APGAR scores [15,16]. Mousavi et al. found that mothers who were indicated for episiotomy had infants with significantly lower APGAR scores at both the first and fifth minutes compared to mothers who were not [17]. Additionally, Gommesen et al. compared women who were diagnosed with third- and fourth-degree perineal lacerations to those who were not [18]. They found that in cases where the infant's birth weight was less than 3000 g and the active birthing process duration was less than three hours, there was a significant decrease in the incidence of EPT laceration.

Considering additional birth-related risk factors that might have distorted the correlation, the current study aimed to identify and investigate risk factors related to the occurrence of extensive perineal lacerations. Third- and fourth-degree EPTs were found to be 1.42% in this study’s population, which is marginally less than the average range of incidence reported in the previous literature [19]. In addition, the hospital from which the data was collected is one of the few hospitals in the city of Riyadh, Saudi Arabia, that still uses forceps as an instrument of delivery. Instrument-assisted deliveries and nulliparity have been identified as risk factors for anal sphincter damage post vaginal delivery, which is consistent with earlier studies [2-3,15-13]. Adjusted for independent covariates, epidural injection in the current study was significantly associated with a decreased incidence of EPTs as compared to other studies where it was not significant or contributed to the occurrence of EPTs [20,21]. It makes the epidural route of anesthesia a protective factor rather than a risk factor for EPTs.

In the current study, a point that was not discussed in the literature is the HCW’s experience in relation to EPTs. Adjusted for independent covariates, deliveries assisted by physicians had a greater incidence of EPT in comparison with midwives-assisted deliveries. Fatal indication (APGAR score) was not of significance to the occurrence of EPTs post or prior model adjustment, which correlates with a previous study by Christianson et al. [13]. Only before the statistical model adjustment of other variables, episiotomy and gestational age were considered risk factors. Moreover, other factors that were not associated with increased or decreased risk of perineal tearing prior and post model adjustment were induction of labor, labor duration, maternal age, and APGAR score.

The small number of instrumental deliveries (vacuum and forceps-assisted deliveries), as well as inherent limitations in a retrospective analysis that prevent control over data quality, are considered limitations of our study. Depending on medical records introduces the possibility of inconsistency in record keeping among different time periods and clinicians. Furthermore, there are some anal sphincter injury potential risk factors, such as perineal body length and station of fetal presentation during the deliveries, that were not available in patients' medical records. Consequently, it was challenging to identify associations since relevant predictors of injury were not recorded in patients' files.

The strengths of our study include the comparison between physicians and midwives, exploring their associations with the occurrence of EPTs. We also included episiotomy as a risk factor and studied its association with EPTs. Because of that, our conclusion is that in vaginal deliveries, obstetric decision-making should give priority to reducing modifiable risk factors for perineal injury. This process involves assessing existing risk factors, such as instrument usage, nulliparity, and a previous episiotomy, and basing decisions on their presence. Considerations may include choosing between vacuum and forceps-assisted delivery, determining the necessity of operative vaginal delivery, and deciding whether to perform an episiotomy. Future studies can improve by implementing more systematic documentation, including detailed dictation and delivery records, and recording all variables associated with the delivery, such as head position and fetal station. A computerized labor and delivery database, like in European studies, would enhance trend analysis and future research. Future studies should investigate more about these potential risk factors, such as maternal height and perineal body length and weight. In the end, future investigations and studies could benefit from patient medical records containing follow-up data regarding both past medical records and maternal symptoms after delivery, including pain, fecal incontinence, and sexual dysfunction that occur in Saudi Arabia, and the type of episiotomy and its associations with EPTs.

## Conclusions

To conclude, by considering the multiple factors discussed earlier in the study, the occurrence of perineal lacerations during childbirth could be prevented. Healthcare providers should take the necessary preventative measures with patients who are deemed at risk of extensive perineal damage. A group of various obstetric factors were associated with third- and fourth-degree perineal tearing. Only four parameters had statistically significant results after adjustment with the other variables, such as nulliparous mothers, instrumental mode of delivery, and epidural usage. Multiparous women had less incidence of extended perineal laceration, and instrumental deliveries have been associated with an increased chance of EPT occurrence. However, epidural anesthesia was a protective factor in this study against the incidence of extensive perineal tearing. Furthermore, as compared to midwives, this study showed that the majority of EPTs occurred when physicians assisted (residents/consultants). Further research, proper documentation, and the development of evidence-based guidelines are needed to improve perineal care and reduce EPT incidence.

## References

[1] Goh R, Goh D, Ellepola H (2018). Perineal tears - a review. Aust J Gen Pract.

[2] Samuelsson E, Ladfors L, Lindblom BG, Hagberg H (2002). A prospective observational study on tears during vaginal delivery: occurrences and risk factors. Acta Obstet Gynecol Scand.

[3] Smith LA, Price N, Simonite V, Burns EE (2013). Incidence of and risk factors for perineal trauma: a prospective observational study. BMC Pregnancy Childbirth.

[4] Edqvist M, Hildingsson I, Mollberg M, Lundgren I, Lindgren H (2017). Midwives' management during the second stage of labor in relation to second-degree tears-an experimental study. Birth.

[5] Jangö H, Langhoff-Roos J, Rosthøj S, Sakse A (2014). Modifiable risk factors of obstetric anal sphincter injury in primiparous women: a population-based cohort study. Am J Obstet Gynecol.

[6] Ramm O, Woo VG, Hung YY, Chen HC, Ritterman Weintraub ML (2018). Risk factors for the development of obstetric anal sphincter injuries in modern obstetric practice. Obstet Gynecol.

[7] Evers EC, Blomquist JL, McDermott KC, Handa VL (2012). Obstetrical anal sphincter laceration and anal incontinence 5-10 years after childbirth. Am J Obstet Gynecol.

[8] Frigerio M, Manodoro S, Bernasconi DP, Verri D, Milani R, Vergani P (2018). Incidence and risk factors of third- and fourth-degree perineal tears in a single Italian scenario. Eur J Obstet Gynecol Reprod Biol.

[9] Shveiky D, Patchen L, Chill HH, Pehlivanova M, Landy HJ (2019). Prevalence and location of obstetric lacerations in adolescent mothers. J Pediatr Adolesc Gynecol.

[10] Chiu K, Mckay E, Fazzari M, Leegant A (2021). Risks and associations of third- and fourth-degree lacerations: an urban single center experience. Female Pelvic Med Reconstr Surg.

[11] Combs CA, Robertson PA, Laros RK (1991). Risk factors for third-degree and fourth-degree perineal lacerations in forceps and vacuum deliveries. Am J Obstet Gynecol.

[12] Peleg D, Kennedy CM, Merrill D, Zlatnik FJ (1999). Risk of repetition of a severe perineal laceration. Obstet Gynecol.

[13] Christianson LM, Bovbjerg VE, McDavitt EC, Hullfish KL (2003). Risk factors for perineal injury during delivery. Am J Obstet Gynecol.

[14] Eskandar O, Shet D (2009). Risk factors for 3rd and 4th degree perineal tear. J Obstet Gynaecol.

[15] Rasouli M, Keramat A, Khosravi A, Mohabatpour Z (2016). Prevalence and factors associated with episiotomy in Shahroud City, northeast of Iran. Int J Women's Health Reprod Sci.

[16] Soleimanzadeh Mousavi SH, Miri M, Farzaneh F (2021). Episiotomy and its complications. Zahedan J Res Med Sci.

[17] Gommesen D, Nohr EA, Drue HC, Qvist N, Rasch V (2019). Obstetric perineal tears: risk factors, wound infection and dehiscence: a prospective cohort study. Arch Gynecol Obstet.

[18] Hsieh WC, Liang CC, Wu D, Chang SD, Chueh HY, Chao AS (2014). Prevalence and contributing factors of severe perineal damage following episiotomy-assisted vaginal delivery. Taiwan J Obstet Gynecol.

[19] Carroll TG, Engelken M, Mosier MC, Nazir N (2003). Epidural analgesia and severe perineal laceration in a community-based obstetric practice. J Am Board Fam Pract.

[20] Howell CJ (2000). Epidural versus non-epidural analgesia for pain relief in labour. Cochrane Database Syst Rev.

[21] Salhan S, Singhal S (2011). Perineal tears. Textbook of Gynecology.

